# Is Brain Dynamics Preserved in the EEG After Automated Artifact Removal? A Validation of the Fingerprint Method and the Automatic Removal of Cardiac Interference Approach Based on Microstate Analysis

**DOI:** 10.3389/fnins.2020.577160

**Published:** 2021-01-12

**Authors:** Gabriella Tamburro, Pierpaolo Croce, Filippo Zappasodi, Silvia Comani

**Affiliations:** ^1^Department of Neuroscience, Imaging and Clinical Sciences, University “G. d’Annunzio” of Chieti-Pescara, Chieti, Italy; ^2^BIND-Behavioral Imaging and Neural Dynamics Center, University “G. d’Annunzio” of Chieti-Pescara, Chieti, Italy; ^3^Institute for Advanced Biomedical Technologies, University “G. d’Annunzio” of Chieti-Pescara, Chieti, Italy

**Keywords:** EEG, artifact removal, ICA, optimized fingerprint method, ARCI approach, microstate analysis

## Abstract

The assessment of a method for removing artifacts from electroencephalography (EEG) datasets often disregard verifying that global brain dynamics is preserved. In this study, we verified that the recently introduced optimized fingerprint method and the automatic removal of cardiac interference (ARCI) approach not only remove physiological artifacts from EEG recordings but also preserve global brain dynamics, as assessed with a new approach based on microstate analysis. We recorded EEG activity with a high-resolution EEG system during two resting-state conditions (eyes open, 25 volunteers, and eyes closed, 26 volunteers) known to exhibit different brain dynamics. After signal decomposition by independent component analysis (ICA), the independent components (ICs) related to eyeblinks, eye movements, myogenic interference, and cardiac electromechanical activity were identified with the optimized fingerprint method and ARCI approach and statistically compared with the outcome of the expert classification of the ICs by visual inspection. Brain dynamics in two different groups of denoised EEG signals, reconstructed after having removed the artifactual ICs identified by either visual inspection or the automated methods, was assessed by calculating microstate topographies, microstate metrics (duration, occurrence, and coverage), and directional predominance (based on transition probabilities). No statistically significant differences between the expert and the automated classification of the artifactual ICs were found (*p* > 0.05). Cronbach’s α values assessed the high test–retest reliability of microstate parameters for EEG datasets denoised by the automated procedure. The total EEG signal variance explained by the sets of global microstate templates was about 80% for all denoised EEG datasets, with no significant differences between groups. For the differently denoised EEG datasets in the two recording conditions, we found that the global microstate templates and the sequences of global microstates were very similar (*p* < 0.01). Descriptive statistics and Cronbach’s α of microstate metrics highlighted no significant differences and excellent consistency between groups (*p* > 0.5). These results confirm the ability of the optimized fingerprint method and the ARCI approach to effectively remove physiological artifacts from EEG recordings while preserving global brain dynamics. They also suggest that microstate analysis could represent a novel approach for assessing the ability of an EEG denoising method to remove artifacts without altering brain dynamics.

## Introduction

The investigation of the human brain function largely relies on electroencephalography (EEG), a technique characterized by an excellent temporal resolution (Niedermeyer and da Silva, 2005) that has recently undergone several technological advances in the electronics and sensor components to enable continuous, out-of-the-lab, and mobile EEG acquisitions (Thompson et al., 2008; Del Percio et al., 2011; De Vos et al., 2011; Lance et al., 2012; Askamp and van Putten, 2014; Liao et al., 2014; Lopez-Gordo et al., 2014; Comani et al., 2015; Fiedler et al., 2015; Michel et al., 2015; di Fronso et al., 2016, 2019; Filho et al., 2016). A drawback of these latest advancements is that they have also enhanced the EEG sensitivity to severe contamination by electrical activity generated outside of cerebral sources, which can seriously affect the analysis and interpretation of EEG signals.

Interference affecting EEG recordings can be of physiological origin, such as eyeblinks, eye movements, myogenic interference due to head or neck muscle contractions, and artifacts related to the electromechanical activity of the heart, but it can also be due to unstable electrode contact or electrical and mechanical interference from nearby instrumentation and power sources (Croft and Barry, 2000, 2002; Lopez-Gordo et al., 2014; Urigüen and Garcia-Zapirain, 2015). Given that visual inspection of EEG data with manual rejection of artifactual data epochs is a subjective method that depends on the expertise of the researcher, is time-consuming, and generally results in a considerable loss of information on brain function, several methods have been proposed to automatically identify and remove artifacts from EEG recordings (see Islam et al., 2016 for a review). Some methods are based on the regression in the time or frequency domain or on adaptive filtering using simultaneously recorded signals of artifactual activity, such as electro-oculogram (EOG) or electrocardiogram (ECG) (Jafarifarmand and Badamchizadeh, 2013; Kumar and Reddy, 2016). The main disadvantage of these methods is that reference signals can still contain information on brain activity (as in the case of EOG); thus, regressing out artifactual activity inevitably involves also the subtraction of a portion of the relevant brain activity from the EEG signals. Another common drawback of regression methods and adaptive filtering is that the recorded artifactual signals are generally suboptimal for describing the artifactual activity because they generally share frequency content with the genuine brain signals (as in the case of ECG), so that these methods are unable to efficiently remove artifacts from EEG recordings while preserving the information on the brain activity (Goncharova et al., 2003). Moreover, clear reference signals cannot be used for some sources of noise such as myogenic activity or noise from external instrumentation, whereas in some newer applications of EEG, such as in sports sciences applications (Stone et al., 2019), the acquisition of reference signals simultaneously with EEG is often problematic. In these cases, to use regression methods or adaptive filtering for artifact removal could become impossible.

Blind source separation (BSS) methods like independent component analysis (ICA) have been successfully applied to identify and remove artifacts from EEG recordings (Jung et al., 2000; Delorme et al., 2007). These approaches are based on the assumption that signals from artifactual sources which are linearly mixed with true brain activity in EEG recordings are statistically independent of neuronal activity. Therefore, the original raw EEG data can be decomposed into a set of statistically independent source signals (independent components—ICs): some ICs contain signals of artifactual origin, whereas the other ICs contain signals originating from brain activity and can be used to reconstruct EEG signals related to brain activity without being affected by interferences of physiological and non-physiological origin. The most common practice for denoising EEG recordings with ICA is that a skilled operator classifies the ICs by visual inspection, rejects the ICs containing artifactual signals, and retains the ICs related to brain activity to reconstruct artifact-free EEG signals. However, visual inspection is a time-consuming process undoubtedly affected by subjective interpretation of the inspected ICs. Therefore, automated procedures for the IC classification are required to overcome these limitations and achieve a more rapid and reliable denoising of EEG recordings. Several methods have been introduced during the last 15 years to address a feasible model-based artifact identification (Barbati et al., 2004; LeVan et al., 2006; Halder et al., 2007; Mantini et al., 2008; Viola et al., 2009; Nolan et al., 2010; Mognon et al., 2011; Winkler et al., 2011; Chaumon et al., 2015; Daly et al., 2015; Frølich et al., 2015; Radüntz et al., 2015, 2017; Chang et al., 2016; Hou et al., 2016; Kilicarslan et al., 2016; Zou et al., 2016; Sreeja et al., 2018; Croce et al., 2019; Guttmann-Flury et al., 2019). However, none of these methods succeeded to combine all desirable properties for a fully automated artifact rejection method: good performance (i.e., high accuracy, sensitivity, and specificity) on major classes of biological artifacts, good generalizability (across sessions, subjects, recording equipment, and sensor layouts), efficiency (low computational cost, potential for online artifact rejection), and transparency (use of explicit and physiologically meaningful EEG features).

Recently, our group developed the fingerprint method for the automated classification and removal of the most ubiquitous physiological artifacts: eyeblinks, eye movements, myogenic interference, and cardiac artifacts (Tamburro et al., 2018). The concept of the fingerprint method was to use a set of 14 spatial, temporal, spectral, and statistical features—which compose the “fingerprint”—to characterize and automatically classify with a machine leaning approach the ICs of artifactual origin separated from an EEG dataset. The original fingerprint method was subsequently optimized to obtain a more efficient and reliable classification of ICs containing eyeblinks, eye movements, and myogenic interference by means of smaller number of features (Stone et al., 2018). Given the poor performance of the fingerprint method in identifying ICs containing cardiac interference, we developed another method, the automatic removal of cardiac interference (ARCI) approach (Tamburro et al., 2019), where new features were introduced to specifically detect and remove the ICs containing cardiac-related artifacts, including pulse-related interferences.

The optimized fingerprint method and the ARCI approach are independent of reference signals (such as EOG or ECG) and use a specific set of features for each artifact type to maximize classification efficiency. A model built for each type of artifact was used to automatically separate artifactual ICs from all other ICs. Both the optimized fingerprint method and the ARCI approach proved to successfully detect ICs related to physiological artifacts with accuracies comparable or even superior to those of other classification methods, independently of the type of EEG system used for the acquisition, the number and layout of electrodes, and the number of separated ICs. Therefore, the optimized fingerprint method and the ARCI approach satisfy the requirements of good performance, good generalizability, efficiency, and transparency. However, an open issue remains whether these methods preserve the original time–frequency content of genuine EEG dynamics without distortions.

Microstate analysis aims at representing the global dynamics of brain activity recorded in the EEG time course as a sequence of a few scalp potential topographies generated by distributed neural pools that are synchronously active and remain stable for short time intervals of approximately 60–120 ms duration (Michel and Koenig, 2018). Such intervals of topographic stability have been referred to as “microstates” (Lehmann et al., 1987, 1998; Pascual-Marqui et al., 1995). Therefore, the brain dynamics included in a given EEG time course can be represented by a non-casual sequence of microstates without any type of *a priori* hypothesis, for example the *a priori* choice of electrodes of interest or specific time intervals or frequency bands (Murray et al., 2008). From this perspective, the ability of microstates to represent the global brain dynamics is higher with respect to classical spectral methods, because microstate analysis preserves the time information that is conversely lost with spectral approaches. In particular, it has been demonstrated that, in healthy adult resting-state studies, most of the variance (about 75–80%) of the EEG signals is explained by sequences of four specific topographies with fixed polarities, arbitrarily labeled as A, B, C, D (Michel and Koenig, 2018). Global descriptors of the time sequence of these four microstates can be obtained by specific metrics, such as mean duration of each microstate, mean number of microstates per second (occurrence), and percentage of coverage of total dynamics, as well as by the transition probabilities among microstates (Michel and Koenig, 2018).

In the present study, we aimed at demonstrating that the removal of physiological artifacts by means of the optimized fingerprint method and the ARCI approach provided artifact-free EEG signals where the retained global brain dynamics was comparable to that retained in the EEG signals where the artifactual components were removed after visual inspection. To this aim, we assessed brain dynamics in the EEG signals denoised with either one of the two approaches by means of microstate analysis. High-density EEG datasets recorded in a group of healthy subjects at rest in eyes open and eyes closed conditions were used for this study. Each EEG dataset was decomposed by means of ICA, and the optimized fingerprint method and the ARCI approach were applied to the separated ICs to classify the ICs containing physiological artifacts. The outcome of the automated ICs classification was compared with the expert classification of the same ICs based on visual inspection. Then, global brain dynamics was assessed in the two types of denoised EEG datasets by means of microstate topographies, metrics, and directional predominance (based on transition probabilities). The outcomes of microstate analysis in the two types of denoised EEG datasets and in the two experimental conditions (eyes open and eyes closed) were compared to assess the efficiency and reliability of the optimized fingerprint method and the ARCI approach to automatically remove physiological artifacts from EEG recordings while preserving the electrophysiological information on brain activity.

## Materials and Methods

### Participants and EEG Recordings

Fifty-one volunteers (39 males, 12 females; mean ± *SD*: 23.8 ± 4.8 years) participated in the study. None of them was under any pharmacological treatment at the time of recordings. The study was approved by the local ethics committee and complied with the ethical standards outlined in the Declaration of Helsinki. Prior to study participation, all volunteers gave their written informed consent.

EEG activity was recorded by a 128-channel system (Electrical Geodesic) with 250 Hz sampling frequency. Impedances were kept below 50 kΩ. EEG acquisitions lasted for 10 min while participants sat on a comfortable armchair at rest. Each volunteer participated in either one of two rest conditions: eyes open (25 volunteers) or eyes closed (26 volunteers).

### Data Analysis

#### EEG Data Preprocessing

Each EEG dataset was filtered with a Butterworth bandpass filter with cutoff frequencies at 0.3 and 70 Hz; a notch filter at 50 Hz was applied to minimize power line interferences. EEG data were visually inspected to exclude EEG channels exhibiting isoelectric saturation or poor scalp-surface contact or excessive noise from further analysis (McMenamin et al., 2010). The EEG segments, where more than 50% of the electrodes exhibited excessive noise during short time intervals, were trimmed from the data. Datasets were then prewhitened by principal components analysis (PCA; Delorme et al., 2007) and decomposed into 50 ICs using the extended Infomax algorithm, which has been proven to better separate signal sources that may exhibit super-Gaussian and sub-Gaussian distributions (Bell and Sejnowski, 1995; Lee et al., 1999). We decided to decompose all datasets into 50 ICs instead of adopting a square decomposition approach because a variable number of bad channels was removed in the individual EEG datasets during the first preprocessing steps. Therefore, a different number of channels was retained for different EEG datasets. To have comparable results across all datasets, we decided to decompose all datasets into an equal number of ICs. Given that the number of retained EEG channels was always greater than 50 and that we had demonstrated in a previous study that artifactual ICs can be successfully identified with the fingerprint method regardless of the decomposition level applied to the analyzed EEG datasets (Tamburro et al., 2018), we decided to decompose each EEG dataset in 50 ICs, which is a decomposition level that can be applied successfully to EEG datasets with 64 channels or more. By doing so, the results obtained in the present study can be considered applicable to a large number of different EEG setups.

All EEG data preprocessing and decomposition were performed using the EEGLAB toolbox (v. 13.6.5b, Delorme and Makeig, 2004).

#### Classification of Artifactual ICs

To assess the effectiveness of the optimized fingerprint method and ARCI approach in removing physiological artifacts without altering the electrophysiological information on the brain activity, the 50 ICs separated from each EEG dataset (both experimental groups: eyes open and eyes closed) were classified with two different approaches: (1) by visual inspection performed by two independent experienced investigators and (2) by means of the optimized fingerprint method and the ARCI approach.

##### Expert classification of artifactual ics by visual inspection

For each EEG dataset, an experienced investigator inspected the time course, topological plot, and power spectrum of each IC of the set of 50 ICs in which the dataset was decomposed. ICs containing eyeblinks or eye movements were labeled as “eye,” ICs containing myogenic interference due to head or neck muscle contractions were labeled as “myogenic,” ICs containing cardiac-related artifacts were labeled as “cardiac,” and ICs related to non-physiological artifacts were labeled as “artifact.” All labels were independently verified by another experienced investigator.

##### Automated classification of artifactual ICs

In the automated classification of artifactual ICs, we applied the fingerprint method optimized for detecting and removing ICs containing eyeblinks, eye movements, and myogenic artifacts (Stone et al., 2018), whereas the ARCI approach (Tamburro et al., 2019) was used to classify the ICs containing cardiac-related artifacts, including pulse interference.

In the optimized fingerprint method, we implemented an optimization procedure that, by means of a genetic algorithm, identified, for each physiological artifact (i.e., eyeblinks, eye movements, and myogenic artifacts), the optimal set of features to obtain an automated non-linear binary support vector machine (SVM) classifier that satisfies three criteria: generalizability, performance, and efficiency (Stone et al., 2018). The three SVM classifiers selected from the optimization procedure (one for each artifact type) were retrained using all available artifactual EEG datasets and composed the final model of the optimized fingerprint method. In this model, the classifier for eyeblinks comprises only four features, the classifier for eye movements comprises 10 features, whereas the classifier for myogenic artifacts comprises all the 14 features originally introduced in the fingerprint method (Tamburro et al., 2018). By sequentially applying these three classifiers to the ICs in which each EEG dataset was decomposed, we classified ICs containing eyeblinks as “eyeblinks,” ICs containing eye movements as “eye movements,” and ICs containing interference from muscle activity as “myogenic.”

The ARCI approach evaluates time and frequency features of the separated ICs to identify ICs that contain electrical cardiac artifacts and/or interference due to the pulsatile activity of the heart (Tamburro et al., 2019). The ARCI approach was applied to the same sets of ICs to classify ICs containing electrical cardiac and cardiovascular artifacts. The ICs classified by ARCI as artifactual contained cardiac-related interference and were labeled as “cardiac.”

#### Removal of Artifactual ICs and Reconstruction of Noise-Free EEG Recordings

The denoising of the EEG datasets (both experimental groups: eyes open and eyes closed) was performed separately for the two approaches used to classify the artifactual ICs.

##### Artifact removal based on visual inspection of the ICs

For each EEG dataset, the ICs labeled by visual inspection as “eye,” “myogenic,” “cardiac,” and “artifact” were disregarded, and the retained ICs, which supposedly contained only signals related to brain activity, were reprojected onto the sensor space to reconstruct noise-free EEG signals. The ensemble of all noise-free EEG datasets reconstructed with this approach composed the reference group of denoised EEG datasets (DNEEG1).

##### Artifact removal based on the automated classification of the ICs

For each EEG dataset, the ICs classified as artifactual by the optimized fingerprint method and the ARCI approach, hence those related to physiological artifacts, were disregarded. Given that the automated denoising methods were not designed to remove non-physiological artifacts, we also removed the ICs labeled as “artifact” by visual inspection (i.e., those ICs containing non-physiological artifacts) in order to make the results of the microstate analysis performed on the differently denoised EEG datasets comparable. Otherwise, the EEG datasets denoised with the automated methods would have retained non-physiological noise that would have altered the results of microstate analysis and therefore hindered a reliable comparison of the ability of the two denoising approaches in preserving global brain dynamics.

For each EEG dataset, the retained ICs were reprojected onto the sensor space to reconstruct noise-free EEG signals. The ensemble of all noise-free EEG datasets reconstructed with this approach composed the test group of denoised EEG datasets (DNEEG2).

#### Validation of the Automated Classification of Artifactual ICs by Means of Microstate Analysis

To assess whether the time–frequency content of genuine brain dynamics was preserved in the noise-free EEG datasets reconstructed after artifact removal with the optimized fingerprint method and the ARCI approach (DNEEG2), we calculated and compared microstates metrics on the DNEEG1 and DNEEG2 datasets (each dataset including both eyes open and eyes closed groups).

##### Microstate analysis

Microstate analysis aims at identifying the dominant topographical configurations (global templates or microstates) that alternate during the EEG time course to depict the ongoing brain dynamics. Through quantitative metrics, it is possible to calculate parameters characterizing the specific sequence of microstates, such as mean duration, coverage, and occurrence frequency of each microstate, and the transition probability from one microstate to another.

**FIGURE 1:**
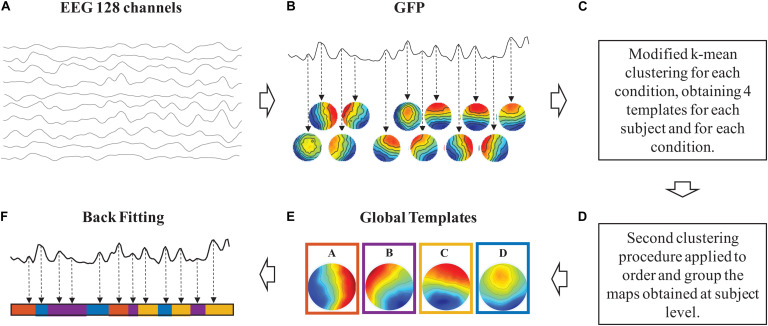
Schematic representation of the sequence of steps taken for the microstate analysis. In clockwise order: samples of 1 s of noise-free EEG signals **(A).** Intervals of stable topographical configurations **(B)**. Identification of the number of dominant microstate templates **(C).** Clustering procedure for the definition of the global microstate templates **(D)**. Identified global templates **(E)**. Backfitting of the global microstate templates to the noise-free EEG signals **(F)**.

The sequence of steps necessary to perform microstate analysis is illustrated in Figure 1. *Step 1*: The intervals of stable topographical configurations are identified in the noise-free EEG recordings (Figures 1A,B). *Step 2*: The global templates of the dominant microstates are calculated for the identified intervals of brain functional stability (Figures 1C–E). *Step 3*: The identified global templates are backfitted to each noise-free EEG dataset to find the specific sequence of microstates on which metrics are calculated (Figure 1F).

*Step 1: Identification of the Intervals of Stable Topographical Configurations*. Each noise-free multichannel EEG dataset from DNEEG1 and DNEEG2 can be viewed as a sequence of instantaneous electric potential distributions over the scalp. To identify intervals of stable topographical configurations (i.e., of stable brain dynamics), we calculated, for each time instant of the EEG time course, the global field power (GFP). GFP is defined as the standard deviation of the EEG signal amplitude across all electrodes at a given time instant and is a descriptor of the potential field strength. In general, a high GFP value is associated with periods of similar potential field distributions, whereas during periods between GFP peaks, the topographic patterns of successive field distributions change rapidly. Therefore, GFP peaks can be considered as corresponding to intervals of highest topographic stability, when the probability to observe a transition to a different (stable) topographical configuration is higher (Murray et al., 2008). For this reason, for each noise-free multichannel EEG dataset from DNEEG1 and DNEEG2, the sequence of instants when GFP peaks occurred were considered the intervals of stable topographical configurations and the corresponding scalp potential distributions were retained for the subsequent steps necessary to identify the microstate templates.

*Step 2: Identification of the Global Templates of the Dominant Microstates*. To identify the global templates representative of the dominant microstates in a group of multichannel EEG recordings, two subsequent clustering procedures were used. The first clustering procedure was applied to individual EEG datasets to identify the optimal number of microstate templates, i.e., the number of microstate templates that explain most of the variance of the EEG signals in individual datasets. The second clustering procedure was applied to all sets of individual microstate templates to identify, by means of a spatial correlation algorithm, the global microstate templates.

This two-step procedure for the identification of the global templates of the dominant microstates was applied to four groups of denoised EEG signals pooled according to the following scheme: (1) EEG signals recorded during eyes open and denoised after expert classification of artifactual ICs (DNEEG1-EO); (2) EEG signals recorded during eyes closed and denoised after expert classification of artifactual ICs (DNEEG1-EC); (3) EEG signals recorded during eyes open and denoised after automated classification of artifactual ICs (DNEEG2-EO); and (4) EEG signals recorded during eyes closed and denoised after automated classification of artifactual ICs (DNEEG2-EC).

##### Identification of the optimal number of microstate templates

The modified version of the clustering *k*-means algorithm, introduced by Pascual-Marqui et al. (1995), was applied to the scalp potential distributions at the instants of GFP peaks obtained for each noise-free multichannel EEG dataset belonging to one of the four groups defined above. For each dataset, the *k*-means algorithm was repeated varying the number of clusters (*k*) from 1 to 12. The optimal number of *k* was identified by applying the Krzanowski–Lai (KL) criterion (see Murray et al., 2008 for details). For each dataset, the centroids of the *k* clusters were then considered the specific set of microstate templates for the given dataset.

##### Identification of the global microstate templates

A set of global microstate templates had to be defined for each of the groups of denoised EEG signals defined above (DNEEG1-EO, DNEEG1-EC, DNEEG2-EO, DNEEG2-EC). For each group, the sets of microstate templates were pooled and an iterative procedure was applied to compose a group of global microstate templates (see Koenig et al., 1999 for details). With this procedure, four sets of *k* global microstate templates were obtained, one for each group of the reconstructed noise-free EEG datasets.

*Step 3: Backfitting of the Global Templates and Microstate Metrics*. For each reconstructed noise-free EEG dataset of each group, the corresponding global microstate templates were backfitted to the EEG signals by calculating the spatial correlation between each global template and the scalp potential distributions at each GFP peak. With this procedure, each EEG time course was represented as a unique sequence of global microstate templates. For each of these sequences, the following metrics were calculated (Lehmann et al., 1987; Brunet et al., 2011):

1. *Mean microstate duration (ms)*: The average duration of each global microstate was calculated as the average time interval during which this microstate remained stable whenever it appeared (Lehmann et al., 1998); the mean microstate duration can be interpreted as an index of stability of the underlying brain dynamics.
2. *Mean microstate occurrence (Hz)*: The frequency with which each global microstate occurred, calculated as the average number of times per second that this microstate became dominant during the EEG time course (Lehmann et al., 1998); the microstate occurrence provides an indication of the tendency of the underlying neural generators to be activated and become dominant.
3. *Mean microstate coverage (%)*: For each global microstate, this metric was calculated as the fraction of the total recording time during which this microstate was dominant (Lehmann et al., 1987).
4. *Transition probabilities (%)*: The probability of transition from one brain state (represented by a global microstate template) to another (represented by another global microstate template) was calculated by counting, for each sequence of global microstates (representing the time course of a noise-free multichannel EEG dataset), the number of transitions from each global microstate template to any other. The occurrences of each transition were then normalized to all microstate template transitions (called transition percentages).
5. *Directional predominance between microstates (%):* The directional predominance between two global microstate templates A and B was defined as the difference between the probability of transition from A to B and the probability of transition from B to A (Lehmann et al., 2005). The directional predominance then quantifies directional asymmetries in the transitions between two global microstate templates. For any given pair of global microstate templates A and B, the directional predominance A↔B can be positive (indicating that there is a higher probability to transit from A to B than from B to A) or negative (indicating that there is a higher probability to transit from B to A than from A to B).

### Statistical Analysis

#### Classification of Artifactual ICs

The similarity between the IC classifications performed by visual inspection and with the optimized fingerprint method and the ARCI approach was assessed with the non-parametric McNemar test for each artifact type and separately for the eyes open and eyes closed conditions. Given that each EEG dataset was decomposed in 50 ICs, the IC classification for each artifact type resulted, for each EEG dataset, in a vector of 50 labels that could be either 1 or 0: 1 identified an artifactual IC and 0 identified a non-artifactual IC. For each artifact type, the vectors of IC labels obtained with the two classification methods (expert and automated) from all decomposed EEG datasets in the two rest conditions were concatenated, resulting in four concatenated label vectors: two vectors for the expert classification (one for eyes open and one for eyes closed) and other two vectors for the automated classification (one for eyes open and one for eyes closed). Given that the expert classification did not differentiate between eyeblinks and eye movements, the label vectors obtained with the automated classification for these two artifacts were merged before building the concatenated label vector.

The non-parametric McNemar test with a correction for continuity was then performed for each rest condition (eyes open and eyes closed) and for each artifact type (i.e., “eye,” “myogenic,” and “cardiac”) on paired concatenated label vectors obtained from the two differently denoised EEG dataset groups. Each concatenated label vector of the eyes open condition contained 1,250 observations (25 eyes open decomposed EEG datasets × 50 ICs), whereas each concatenated label vector of the eyes closed condition contained 1,300 observations (26 eyes closed decomposed EEG datasets × 50 ICs). The null hypothesis of the test was that there was no difference between paired concatenated label vectors. The significance level was set at 0.05.

#### Similarity of Global Microstate Templates Across Groups

First, we quantified the total EEG signal variance explained by the four sets of global microstate templates, and then verified, by means of a two-tailed paired *t*-test, that no significant differences existed between groups (DNEEG1-EO vs. DNEEG2-EO and DNEEG1-EC vs. DNEEG2-EC).

Second, the similarity between the sets of global microstate templates extracted from the EEG datasets denoised by expert classification and by automated classification (DNEEG1 and DNEEG2) was statistically assessed by means of the topographical analysis of variance (TANOVA, Koenig et al., 2014) separately for the two rest conditions (eyes open and eyes closed): Comparisons were performed between the sets of global microstate templates of DNEEG1-EO and DNEEG2-EO and between the sets of global microstate templates of DNEEG1-EC and DNEEG2-EC.

TANOVA is based on the evaluation of an effect size between groups. We quantified the effect size by computing the global dissimilarity (GD) between two global microstate templates as:

$$G\,D_{u,v}=\sqrt{\frac{1}{N}\,\sum_{i=1}^{N}{\left(\frac{u_{i}}{G\,F\,P_{u}}-\frac{v_{i}}{G\,F\,P_{v}}\right)}^{2}}$$

where *u*_*i*_ and *v*_*i*_ are the electric potentials of the *i*_*th*_ electrode in the microstate templates *u* and *v*, respectively; *GFP*_*u*_ and *GFP*_*v*_ are the global field powers of the microstate templates (*u* and *v*); and *N* is the number of electrodes (hence of electric potential values in each microstate template). *GD*_*u*,*v*_ can vary between 0 and 2: 0 indicates that the compared microstate templates are identical, whereas 2 indicates that these two microstate templates are opposite (i.e., have reversed polarity).

Given that we are interested in assessing the similarity between global microstate templates of the same type, we calculated the statistical significance of the *GD*_*u*,*v*_ values obtained for pairs of global microstate templates of the same type from DNEEG1-EO and DNEEG2-EO and for pairs of global microstate templates of the same type from DNEEG1-EC and DNEEG2-EC. To do so, the *GD*_*u*,*v*_ value obtained for a pair of global microstate templates was compared with a reference random distribution of GD values between microstate templates of individual noise-free EEG datasets from DNEEG1-EO and DNEEG2-EO (or from DNEEG1-EC and DNEEG2-EC) under the null hypothesis that microstate templates with the same label were different. To build this random distribution (simulated effect size distribution), GD was computed for pairs of microstate templates after randomly shuffling individual microstate templates between groups for a sufficient number of times (we used 10,000 random permutations) (Koenig and IVIeIle-Garcia, 2009). Then, the percentage of *GD*_*u*,*v*_ values in the random distribution that were greater than the *GD*_*u*,*v*_ value obtained for the corresponding pair of global microstate templates gave us the *p*-value associated with that specific *GD*_*u*,*v*_ value. The significance level was set at 0.01. Therefore, *p*-values lower than 0.01 indicated that we should accept the null hypothesis and that the two compared microstate templates could be considered statistically identical.

#### Statistical Assessment of Microstate Metrics

The metrics calculated on the sequences of global microstate templates obtained for each noise-free multichannel EEG dataset after backfitting the global microstate templates to the EEG signals (mean microstate duration, mean microstate occurrence, mean microstate coverage, and directional predominance between pairs of microstate templates) were statistically compared between groups (DNEEG1-EO vs. DNEEG2-EO and DNEEG1-EC vs. DNEEG2-EC) by means of a two-tailed paired sample *t*-test with the significance level set at 0.05.

To assess the level of consistency between the microstate parameters extracted from EEG datasets denoised by means of the two approaches (visual inspection and automated classification), we calculated Cronbach’s α between the microstate parameters (i.e., duration, occurrence, coverage, directional predominance) obtained from the differently denoised datasets in both eyes closed and eyes open conditions (DNEEG1-EO vs. DNEEG2-EO and DNEEG1-EC vs. DNEEG2-EC). Cronbach’s α is a relative measure of test–retest reliability, hence a measure of between-subject variance relative to within-subject variance. Values greater than 0.70 indicate high reliability.

#### Test–Retest on Microstate Metrics to Validate Automated Denoising

To determine the test–retest reliability of the automated denoising procedure (optimized fingerprint method and ARCI approach), both eyes closed and eyes open EEG datasets were split in two subsets (first and second half), each consisting of 5 min of recording. The whole analysis pipeline (ICA decomposition, automated classification by means of the optimized fingerprint method and ARCI approach, denoising, microstate extraction, backfitting, microstate parameter calculation) was applied to each data subset. Then, Cronbach’s α between the microstate parameters obtained for the two subsets was calculated.

## Results

### Classification of Artifactual ICs

The non-parametric McNemar test performed to assess differences between the two classification methods yielded non-significant differences in the two rest conditions for all types of artifact (i.e., “eye” and “cardiac”). It is worth noting that both the expert classification and the optimized fingerprint method did not identify any “myogenic” artifactual IC; thus, no statistical assessment of these classifications was performed. The results of the non-parametric tests on the “eye” and “cardiac” artifactual ICs are reported in Table 1. The *p*-values were always much greater than 0.05, indicating that no significant differences between the expert IC classification performed by visual inspection and the IC classification performed with the optimized fingerprint method and the ARCI approach could be observed.

**TABLE 1:** Comparison of the outcome of the two IC classification approaches.

**Rest condition**	**Artifact type**	**Expert classification**		**Automated classification**		***χ*^2^**	***p***
		**Non-art. ICs (%)**	**Art. ICs (%)**	**Non-art. ICs (%)**	**Art. ICs (%)**		
Eyes open	Eye	93.8	6.2	94.6	5.4	2.25	0.134
	Cardiac	98.2	1.8	98.4	1.6	0.07	0.789
Eyes closed	Eye	95.2	4.8	95.2	4.1	1.16	0.281
	Cardiac	98.0	2.0	97.8	2.2	0.05	0.823

*For each rest condition (eyes open and eyes closed) and for each artifact type (“eye” and “cardiac”), we provide the following information: Percentages of ICs classified as non-artifactual and artifactual for the EEG datasets denoised by visual inspection (expert classification of ICs) and by means of the optimized fingerprint method and the ARCI approach (automated classification of ICs); McNemar test value (χ^2^); significance level (p).*

### Microstate Analysis

#### Microstate Clustering Results

For each group of denoised EEG datasets (DNEEG1-EO, DNEEG1-EC, DNEEG2-EO, and DNEEG2-EC), the KL criterion identified four as the optimal number of cluster centroids. Therefore, for each of these groups, we retained four global microstate templates (labeled A, B, C, and D) for further analysis. For all groups, the global microstate A exhibits a left–right orientation of the scalp potential field, the global microstate B exhibits a right–left orientation of the scalp potential field, the global microstate C exhibits an anterior–posterior orientation of the scalp potential field, and the global microstate D exhibits a maximum of the scalp potential field in the central frontal region. The total EEG signal variance explained by the four sets of global microstate templates is as follows: 80 ± 1% for DNEEG1-EO, 81 ± 1% for DNEEG1-EC, 81 ± 1% for DNEEG2-EO, and 79 ± 1.5% for DNEEG2-EC. The two-tailed paired *t*-test assessed no significant differences between groups (DNEEG1-EO vs. DNEEG2-EO and DNEEG1-EC vs. DNEEG2-EC; significance level set at 0.5; *p* = 0.69 for DNEEG1-EO vs. DNEEG2-EO; *p* = 0.62 for DNEEG1-EC vs. DNEEG2-EC).

#### Similarity of Global Microstate Templates Across Groups

**FIGURE 2:**
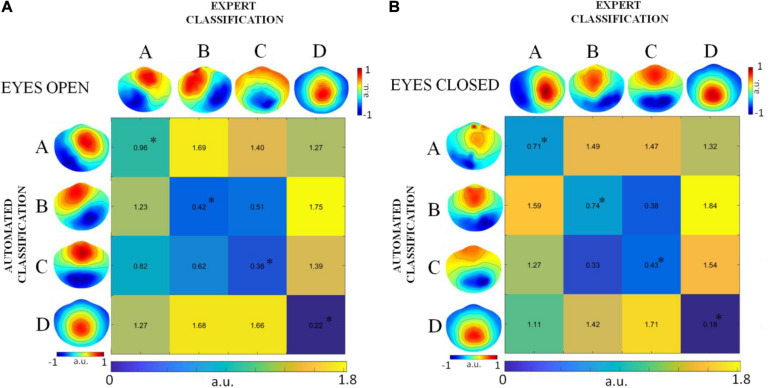
GD values obtained for paired global microstate templates in the eyes open condition **(A)** and in the eyes closed condition **(B)**. Diagonal values correspond to the GD between global microstate templates of the same type obtained from DNEEG1-EO and DNEEG2-EO **(A)** and from DNEEG1-EC and DNEEG2-EC **(B)**. The asterisk indicates GD values for which *p* < 0.01.

The similarity between pairs of global microstate templates from the EEG datasets denoised by expert classification and by automated classification was assessed by means of global dissimilarity (GD) separately for the two rest conditions (DNEEG1-EO vs. DNEEG2-EO and DNEEG1-EC vs. DNEEG2-EC). The results obtained are summarized in Figure 2A refers to the eyes open condition, whereas Figure 2B refers to the eyes closed condition. The GD values in the diagonal of each matrix in Figure 2 correspond to the GD values between two global microstate templates of the same type obtained from either DNEEG1-EO and DNEEG2-EO or DNEEG1-EC and DNEEG2-EC. These GD values permit to assess whether the global microstate templates obtained from DNEEG2-EO (DNEEG2-EC) were similar to the global microstate templates obtained from DNEEG1-EO (DNEEG1-EC). The GD values in the diagonals were the lowest among all GD values in both matrices: They ranged from 0.22 to 0.96 for the eyes open condition and from 0.18 to 0.74 for the eyes closed condition, hence suggesting that the global microstate templates of the same type were very similar in the two groups. The *p*-values associated with these GD values were always lower than 0.01, indicating that the four global microstate templates obtained for DNEEG1-EO and DNEEG2-EO and for DNEEG1-EC and DNEEG2-EC did not significantly differ from each other.

#### Backfitting of Global Microstate Templates and Statistical Assessment of Microstate Metrics

**FIGURE 3:**
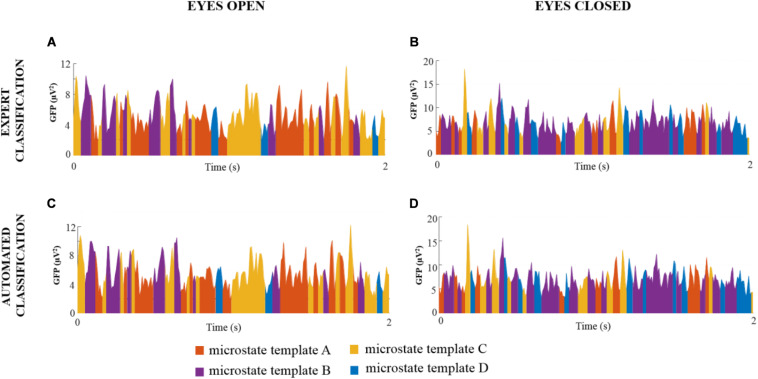
Examples of sequences of global microstate templates for a representative EEG dataset (subject 1 for each condition) denoised after expert and automated classification of the ICs. The *X*-axis represents the time (s) and the *Y-*axis represents the GFP values of the EEG signal. Each GFP sample is assigned to one global microstate template, thus resulting in a sequence of four different colors: orange indicates the global microstate template A, purple indicates the global microstate template B, yellow indicates the global microstate template C, and blue indicates the global microstate template D.

For each noise-free multichannel EEG dataset of DNEEG1-EO, DNEEG2-EO, DNEEG1-EC, and DNEEG2-EC, the four global microstate templates were backfitted to the EEG signals to obtain a sequence of global microstates representative of the dominant stable brain states. For all multichannel EEG datasets, the sequences of global microstates were very similar for the same EEG dataset denoised after the expert classifications of the ICs and after the automated classifications of the ICs. Examples of sequences of global microstate templates are provided in Figure 3.

**FIGURE 4:**
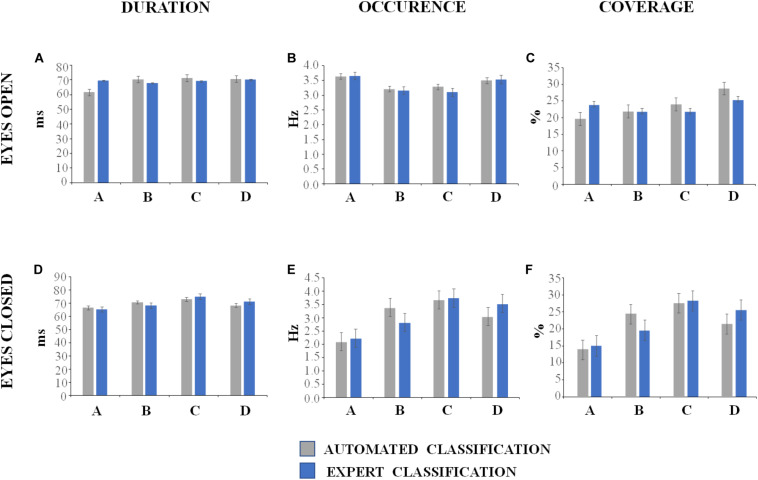
Descriptive statistics of microstate metrics (mean values with standard errors). The labels of the global microstate templates are given in the *X*-axes of **(A–F)**.

**FIGURE 5:**
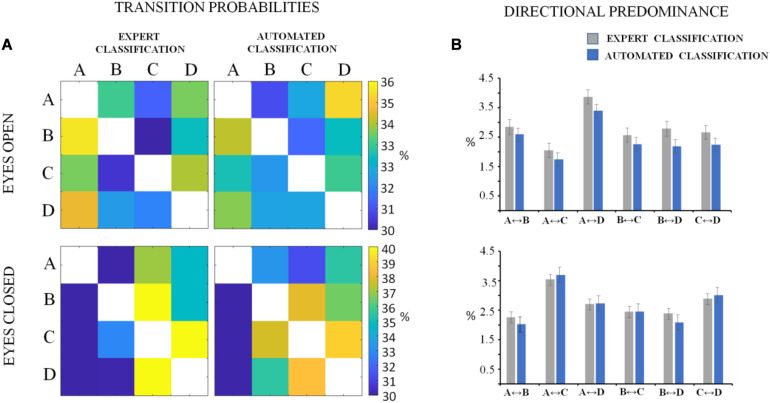
Matrices of transition probabilities in the eyes open and eyes closed conditions **(A)**. The *ij* element of the matrix corresponds to the probability to transit from microstate *i* to microstate *j*. In **(B)**, the descriptive statistics of absolute values of directional predominance (mean values with standard errors) are shown. The labels of the directional predominance types are given in the *X*-axis.

The descriptive statistics of the microstate metrics on the sequences of global microstate templates between groups (DNEEG1-EO vs. DNEEG2-EO and DNEEG1-EC vs. DNEEG2-EC) are given in Figures 4, 5. Given that we retained four global microstate templates, 12 different transitions could occur between global microstate templates (i.e., A→B, A→C, A→D, B→A, B→C, B→D, C→A, C→B, C→D, D→A, D→B, D→C, Figure 5A), from which six types of directional predominance could be identified (A↔B, A↔C, A↔D, B↔C, B↔D, C↔D, Figure 5B). The two-tailed paired sample *t*-test performed for each metric (i.e., mean microstate duration, mean microstate occurrence, mean microstate coverage, transition probabilities, and directional predominance) between groups (DNEEG1-EO vs. DNEEG2-EO and DNEEG1-EC vs. DNEEG2-EC) gave no significant differences between groups: *p*-values were always greater than 0.5.

Consistency between the microstate parameters extracted from the multichannel EEG datasets denoised by means of the two methods (expert classification of ICs vs. automated classification of ICs) is shown in Table 2. Very high levels of Cronbach’s α, always higher than 0.99, were obtained for all microstate parameters in both eyes closed and eyes open conditions.

**TABLE 2:** Consistency between microstate parameters extracted from multichannel EEG dataset denoised by means of the visual inspection approach and the automated approach.

	**A**	**B**	**C**	**D**		

	***Duration***					
EC	0.996	0.996	0.998	0.999		
EO	0.996	0.999	0.998	0.999		

	***Occurrence***					

EC	0.998	0.998	0.997	0.999		
EO	0.997	0.999	0.999	0.998		

	***Coverage***					

EC	0.997	0.995	0.998	0.998		
EO	0.997	0.999	0.999	0.999		

	***Directional predominance***					

	**A↔B**	**A↔C**	**A↔D**	**B↔C**	**B↔D**	**C↔D**

EC	0.995	0.998	0.996	0.997	0.996	0.998
EO	0.998	0.993	0.997	0.999	0.998	0.996

*For each rest condition (eyes open: EO and eyes closed: EC), we indicate the Cronbach’s α between microstate parameters obtained from the two datasets.*

A high test–retest reliability was obtained between microstate parameters of the EEG datasets denoised by the automated procedure (ARCI and the optimized fingerprint method). The consistency between the microstate parameters extracted from the first half and the second half of the multichannel EEG dataset denoised by means of the automated procedure is shown in Table 3. Mean Cronbach’s α values across microstate templates and conditions are 0.882, 0.894, 0.894, and 0.824, for duration, occurrence, coverage, and directional predominance, respectively.

**TABLE 3:** Test–retest reliability of microstate parameters extracted from the multichannel EEG dataset denoised by means of the automated approach (ARCI and optimized fingerprint method).

	**A**	**B**	**l**	**D**		

	***Duration***					
EC	0.801	0.938	0.984	0.939		
EO	0.915	0.894	0.868	0.727		

	***Occurrence***					

EC	0.898	0.868	0.926	0.911		
EO	0.824	0.900	0.884	0.937		

	***Coverage***					

EC	0.875	0.889	0.972	0.901		
EO	0.861	0.894	0.878	0.882		

	***Directional predominance***					

	**A↔B**	**A↔C**	**A↔D**	**B↔C**	**B↔D**	**C↔D**

EC	0.860	0.953	0.750	0.847	0.876	0.957
EO	0.703	0.813	0.744	0.756	0.825	0.799

*For each rest condition (eyes open: EO and eyes closed: EC), we indicate Cronbach’s α between microstate parameters of the first half and the second half of the EEG recordings.*

## Discussion

The availability of a fully automated method that, by means of sets of specific features, is able to identify artifactual ICs and, therefore, remove physiological artifacts from EEG recordings of various types results in the possibility to apply EEG in a variety of acquisition conditions and in a great simplification of both the EEG recording setting and the subsequent analysis (Melissant et al., 2005; De Vos et al., 2011; Daly et al., 2015; di Fronso et al., 2018; Stone et al., 2019). The availability of such a method would also guarantee a standardization of the EEG preprocessing step, independent from the operator. In this study, we demonstrated the ability of the optimized fingerprint method and the ARCI approach to identify and remove physiological artifacts while preserving brain dynamics, assessed by means of microstate analysis.

Our results highlighted two main aspects. The first aspect regards the confirmation of our previous results (Stone et al., 2018; Tamburro et al., 2018, 2019) on the suitability and efficacy of the optimized fingerprint method and the ARCI approach in identifying physiological artifactual ICs. Indeed, in a sample of healthy adults at rest in the two physiological conditions of eyes open and eyes closed, we did not find any significant differences between the IC classifications performed by expert visual inspection and by the automated methods. When comparing the outcome of the ARCI approach and the optimized fingerprint method with the classifications made by experienced investigators (see Table 1), the differences of percentages of ICs classified as artifactual ICs related to cardiac interference and to ocular activity were below 0.4 and 1.4%, respectively. Other studies addressed the issue of the use of features based on spectral, spatial, temporal, and statistical characteristics of artifactual signals to identify artifactual ICs and remove non-cerebral activity from EEG datasets (for a review, see Urigüen and Garcia-Zapirain, 2015). With respect to prior methods, the added value of the optimized fingerprint method and the ARCI approach relies on their ability of combining multiple signal features and selecting their best combination to detect specific artifacts based on actual performance (Stone et al., 2018; Tamburro et al., 2019). In other words, the combination of a specific set of the most relevant features for each type of physiological artifact facilitates the identification of these artifacts and their subsequent removal while reducing their computational cost.

The second main aspect of our study regards the use of microstate analysis to assess the performance—and demonstrate the suitability and reliability—of the tested denoising methods in removing physiological artifacts while preserving global brain dynamics. To our knowledge, this is an absolute novelty and a step forward in the assessment of automated methods designed to eliminate physiological interference from EEG recordings. Usually, the validation of an artifact removal method is performed by comparing the denoised signal (i.e., the original raw signal minus the estimated artifactual activity) with the original noiseless signal (i.e., the signal of brain origin). This approach implies that the original brain signal should be known *a priori*, which is of course not possible, and this *a priori* knowledge is usually achieved by means of simulated data. Nevertheless, it would be important to assess the reliability of denoising methods also with real EEG data (Urigüen and Garcia-Zapirain, 2015). The calculation of the correlation between the corrected EEG data and the reference artifactual signal(s) (Croft et al., 2005; Pham et al., 2011; Sweeney et al., 2012) in both the time and frequency domains is an indirect way to quantitatively evaluate the performance of an artifact removal method. However, this approach has two main limitations: one regards the need to monitor the artifactual activity by recording the reference signal(s) (EOG, EMG, ECG, etc.) together with the EEG signals; the other limitation is related to the use of a global measure (i.e., the correlation between time series) to assess the performance of the denoising method: in fact, the correlation does not account for the dynamics of cerebral activity and is not suitable to quantify possible loss of electrophysiological information during the artifact removal process. Other global metrics, such as mean power or maximum amplitude, kurtosis, and skewness of the amplitude of resting-state background rhythms, have been proposed to characterize the behavior of brain EEG signals and artifactual activity in order to evaluate the performance of methods designed to remove artifactual ICs (Daly et al., 2015; Mannan et al., 2018). In some studies, statistical features characterizing the artifactual ICs were quantified and used to check whether the cleaned EEG signals resembled noise-free EEG signals (Delorme et al., 2007; Mognon et al., 2011). Further validation approaches compared the brain activity or EEG topography before and after artifact removal by means of visual inspection of one or more time intervals or frequency bands in one or more channels grouped in regions of interest (De Clercq et al., 2006; Crespo-Garcia et al., 2008; Romero et al., 2008, 2009; De Vos et al., 2010; Kirkove et al., 2014; Santillán-Guzmán et al., 2017). However, all these approaches did not consider the dynamics of brain activity and were thus prone to possible loss of time information in the reconstructed brain signals.

Conversely, the validation approach used in the present study considers both the global information coming from the topography of the EEG signals and the temporal dynamics of sequences of neural activity. By means of microstate analysis, the ongoing EEG time course was represented by a non-casual sequence of semistable scalp potential topographies, and specific characteristics of this sequence were assessed by global metrics that are good global descriptors of brain dynamics. Therefore, microstate analysis is an optimal approach to demonstrate whether a denoising method can identify and remove physiological artifacts from EEG recordings without distorting brain dynamics. By applying this validation approach, we firstly confirmed that the automated approaches (ARCI and optimized fingerprint methods) have high test–retest reliability, in agreement with the results on validation of these methods previously obtained by means of other approaches (Stone et al., 2018; Tamburro et al., 2019). Moreover, we verified that there were no statistical differences between the outcome of microstate analysis in the EEG datasets denoised by means of the optimized fingerprint method and the ARCI approach and by means of visual inspection. We also demonstrated an excellent consistency between the two denoising approaches, since the values of Cronbach’s α between the microstate parameters obtained by the two denoised EEG datasets are always above 0.90 (see Table 2). Furthermore, we verified that the two denoising approaches (the approach based on the expert classification of the artifactual ICs and the one based on the automated classification of the artifactual ICs) had equivalent performances in rest EEG datasets recorded with eyes closed and with eyes open, which are very different physiological conditions for which it is known that differences in spectral content and brain dynamics exist (Niedermeyer and da Silva, 2005). Another important aspect of the use of microstate analysis to validate a denoising method is that the outcome of microstate analysis provides information on the efficiency and reliability of the denoising method at different levels. First, dominant scalp potential distributions are represented by the global microstate template topographies. We verified that the global microstate templates of the same type were statistically identical in the two groups of denoised EEG data and that they were very similar to those found in resting-state EEG activity in healthy adults and labeled A, B, C, and D (Lehmann et al., 1987; Michel and Koenig, 2018). Second, we found that when these four topographies were backfitted to the denoised EEG data, they accounted for about 80% of the variance in both cases, without differences. This result indicates the ability of the optimized fingerprint method and the ARCI approach to effectively identify and remove all physiological artifacts without altering the information on brain dynamics. Moreover, the sequences of global microstates were very similar for the same EEG datasets denoised after the expert and the automated classifications of the ICs, highlighting a lack of distortion of the EEG dynamics after the application of the automated denoising methods. Third, the metrics describing the microstate dynamics (duration, occurrence, coverage, and directional predominance) were equivalent in the EEG datasets denoised after the expert and the automated classification of the ICs, thus confirming that the information on the neural activity dynamics was not altered. In particular, the microstate duration quantifies the interval of stability of neural activity patterns underlying specific microstates and, thus, reflects the stability of the activity of these neural assemblies (Michel and Koenig, 2018). The occurrence, i.e., the average number of times per second that a given microstate occurs, quantifies the tendency of its underlying neural generators to be activated (Khanna et al., 2015). The percentage of total time that a microstate is dominant indicates the relative presence of this microstate, thus the relative presence of the corresponding pool of active neural generators with respect to the others (Michel and Koenig, 2018). Finally, the directional predominance quantifies the probability of transition between microstates, thus the probability associated with a specific evolution of brain dynamics. The fact that we found high consistency and no statistically significant differences in the microstate duration, occurrence, coverage, and directional predominance when comparing the microstate metrics obtained for the EEG datasets denoised after the expert and the automated classification of the ICs demonstrates that the application of the optimized fingerprint method and the ARCI approach to rest EEG recordings succeeded to remove the physiological artifacts without altering the brain dynamics contained in the EEG signals.

The importance of removing artifacts from EEG recordings to obtain reliable information on brain activity and brain dynamics in various experimental and clinical conditions does not need to be demonstrated. What still needs to be assessed is the reliability of the denoising methods proposed during the last decade and their ability to preserve brain dynamics. With the study described herein, we believe to have made a step forward in the general effort to validate a denoising method (or a group of denoising methods) not only with respect to its (their) ability in identifying and removing physiological artifacts from EEG recordings but mainly in verifying that they do not alter the global brain dynamics contained in the EEG signals. Microstate analysis, by its ability to quantify the state and evolution of brain activity, is a powerful means of verification that brain dynamics has not been altered by the application of denoising methods. We think that the results reported herein allow us to conclude that the global brain dynamics retained in EEG signals after removing physiological artifacts with the optimized fingerprint method and the ARCI approach is comparable to the global brain dynamics retained in EEG signals denoised by visual inspection. Furthermore, we think that we have outlined a new approach based on microstate analysis that is suitable to assess whether automated denoising methods are effective not only in removing artifacts from EEG recordings but also in preserving brain dynamics.

## Data Availability Statement

The raw data supporting the conclusions of this article will be made available by the authors, without undue reservation, to any qualified researcher.

## Ethics Statement

The studies involving human participants were reviewed and approved by the Ethics Committee of the University “G. d’Annunzio” of Chieti-Pescara (Italy). The patients/participants provided their written informed consent to participate in this study.

## Author Contributions

GT, PC, FZ, and SC conceived and designed the study and wrote, revised, and edited the manuscript. FZ and PC ran the experiments. GT and PC analyzed the data. All authors contributed to the article and approved the submitted version.

## Conflict of Interest

The authors declare that the research was conducted in the absence of any commercial or financial relationships that could be construed as a potential conflict of interest.
